# Bringing Traditional Medicine into the Health Humanities Classroom with Kali Fajardo-Anstine’s “Remedies”

**DOI:** 10.1007/s10912-024-09906-5

**Published:** 2024-10-15

**Authors:** Jess Libow, Lindsey Grubbs

**Affiliations:** 1 Writing Program, Haverford College, Philadelphia, PA USA; 2 Department of Bioethics, Case Western Reserve University, Cleveland, OH USA

**Keywords:** Undergraduate education, Cultural humility, Traditional medicine, Biomedicine, Chicana literature

## Abstract

In this essay, we recommend Kali Fajardo-Anstine’s short story “Remedies” (2019) for inclusion on health humanities syllabi based on our experiences teaching it at two undergraduate institutions. The story is drawn from *Sabrina & Corina*, Fajardo-Anstine’s award-winning book of short stories about Chicana and Indigenous women in Colorado, but is available for free online, making it highly accessible for students. “Remedies” is narrated by Clarisa, who turns to her great-grandmother Estrella for the traditional knowledge that ultimately cures her family’s recurrent outbreaks of lice. As a health narrative that centers familial and cultural healing practices, “Remedies” offers a much needed counterpart to the biomedical frameworks that tend to dominate health humanities syllabi and curricula. At the same time that it illuminates the physical and emotional efficacy of such practices, “Remedies” rejects a binary that pits them against biomedicine, offering a complex portrait of how various members of a family integrate traditional and biomedical approaches to health. We discuss how themes related to familial and cultural healing practices are developed in the story and introduce our approach to initiating productive conversations about the relationship between traditional healing and biomedicine in our classrooms.

In the opening lines of Kali Fajardo-Anstine’s short story “Remedies” (2019a, b), the narrator, Clarisa, demonstrates the vast health knowledge she has inherited from her great-grandmother: “A dermatologist with a can of liquid nitrogen can remove a wart in four to five seconds. I can remove one overnight with a clove of garlic and a Band-Aid” (70). The list goes on: “If you have a stomachache, drink chamomile tea with honey…if you have a headache, put slices of potato at your temples…if you have a cold or a broken heart, drink a warm cup of atole made only with blue corn” (70). In Fajardo-Anstine’s story, healing is not the province of clinicians alone, and biomedicine is not the only resource for alleviating bodily suffering. Rather, “Remedies” highlights approaches to health grounded in family history and cultural heritage. For both authors of this essay, discussions of the text in undergraduate health humanities classrooms prompted insightful conversations about the relationship between traditional and biomedical perspectives on health. In what follows, we describe how this theme is developed in the story and reflect on the strategies we used to engage students in this discussion.

One of us first assigned “Remedies” while revising a health humanities syllabus at a public university federally designated as a Hispanic Serving Institution and Asian American and Pacific Islander Serving Institution. Around two-thirds of students were first-generation college students, and around half were Pell Grant eligible. The goals of this revision were to eliminate textbook costs and incorporate readings that reflected the diversity of the student body. “Remedies” helped accomplish both: it was freely available online through *Electric Literature*, and was drawn from a story collection, *Sabrina & Corina* (2019), in which Fajardo-Anstine depicts the lives of Chicana and Indigenous women in Colorado. Students enjoyed the story and reflected thoughtfully on its themes. For these reasons, it was recommended to the other author of this essay, who teaches a first-year health humanities seminar at a liberal arts college and had a similar experience. We hope others will consider including it in their health humanities curricula.

“Remedies” is narrated by Clarisa, who recalls the head lice that plagued her family during her childhood. The lice appear when her mother, Millie, starts hosting visits from Clarisa’s half-brother Harrison, the son of her estranged white father and another woman. While Millie’s motivations are not explicit, they seem to include pity for Harrison and a desire to heal the wounds left by Clarisa’s father. Clarisa, though, sees Harrison as an unwelcome pest. Even as Millie is determined to care for the neglected boy and manage the infestation herself, the lice persist, ultimately driving Clarisa to ask her great-grandmother, who she calls “Grandma Estrella,” for help. There are no biomedical healthcare providers depicted in “Remedies.” Rather, Grandma Estrella is the expert healer to whom Clarisa turns.

“Remedies” participates in what Amanda V. Ellis (2021) and Julie Avril Minich (2023) identify as a robust tradition of Latinx and Chicanx cultural expression representing traditional, community-based healers. Such works offer a necessary counterpart to biomedical frameworks that dominate much required coursework for students pursuing health professions—including health humanities courses focused on texts written by or about clinicians or that center patients’ encounters with Western biomedicine. Traditional healing practices that depart from and even contradict biomedical models remain crucial to many U.S. residents’ understandings of health (Baer 2001). Incorporating texts like “Remedies” can challenge assumptions that such practices are a “problem” for providers to navigate in patient interactions and instead figure traditional healing as a form of “decolonial medicine” that productively challenges tenets of biomedicine. In particular, it “deconstructs dualistic constructions of personhood” that separate mind and body, “restores our attention to holistic health, recalls non-Western bodies of knowledge about health, and treats disease” (Ellis 2021, 7). Existing scholarship (Andrews et al. 2013; Engebretson 1994; Kirmayer 2004; Miville and Hill 2020) has demonstrated the importance of biomedical providers developing an understanding of diverse healing traditions, and Desmarais and Robbins (2019) have identified health humanities classrooms as ideal sites for this work. Indeed, some argue that coordinated efforts between traditional healers and biomedical providers might improve the quality of care for Latinx communities in the face of health disparities (Cruz et al. 2022; Mellinger 2022). “Remedies” emphasizes the crucial role such practices play in many patients’—and providers’—understandings of health.

The approach to healing depicted in “Remedies” integrates physical and spiritual well-being. Latin American *curanderos/as* understand health not only as physical or mental, but also as spiritual (Trotter and Chavira, 2011; Maduro 1983). Ellis (2021), who writes about her own grandmother’s work as a *sobadora* (massage practitioner), uses the term “mindbodyspirit” to illustrate how deeply intertwined these are for traditional Chicanx healers (4). For example, to treat lice, Grandma Estrella creates “a liquid made from something called neem that had a thick rootlike stench” in the “metal pot…normally used for menudo” and pours the mixture over each person’s scalp (83). The treatment takes place in her home, where she nourishes her family. Similarly, after ridding Millie of lice, Grandma Estrella also absolves her of responsibility for her former partner, a man she has long viewed as a parasite himself. “That man and his choices are behind you,” she declares, her hands on Millie’s freshly clean head (84). As such, “Remedies” situates healing as part of what Ellis (2021) calls “everyday woman-of-color folk knowledge and folk practice” (8).

“Remedies” also illustrates the collectivity undergirding many traditional models of health. Grandma Estrella brings Clarisa into the healing process by having her pour the remedy over Harrison’s scalp. As she does so, Clarisa’s attitude towards him shifts. No longer resentful of his presence, she sees “for the first time how incredibly scabbed and bitten he was” and finds herself reassuring him that relief is imminent (84). By participating in healing, Clarisa attends to Harrison’s spiritual as well as physical suffering—just as Grandma Estrella does Millie’s. Indeed, throughout the story, Clarisa shows how healing preserves generational knowledge. Just as Grandma Estrella taught Clarisa to treat a variety of maladies, so too did Estrella “learn[] from her own grandma on their pueblo in northern New Mexico” (70). Estrella continues to play this role even after she’s gone. The adult Clarisa recalls that, “Before Great-Grandma died, she gave me a booklet of everything she knew. Inside, with an unsteady hand she had drawn pictures of plants, and beneath them, their Spanish names, their scientific names, and just for me, their English names” (85). The booklet preserves traditional knowledge and translates it for the English-speaking Clarisa. By depicting Clarisa’s development from patient to healer, “Remedies” illustrates what Minich (2023) has termed a Latinx “politics of radical health” that rejects the “purely individual, medical” notions of health that are too often weaponized against communities of color by instead highlighting “communal responsibility” for well-being (3).

While the story incorporates communal care and family legacies, it features three clearly individuated women who engage with their cultural legacy in distinct ways. Fajardo-Anstine has spoken about her goal of representing mixed-race Latinas, who she notes are “often…invisible in the greater Latinx narrative,” by “creat[ing]…very individualistic, very human” characters (Luna 2019). “Remedies” illuminates the complex identities and experiences people bring to therapeutic work. Grandma Estrella, for example, is judgmental of Harrison’s “dirtiness” due to having been called “a dirty Mexican” herself as a child. By rendering traditional practices as individualistic, “Remedies” refuse to romanticize or stereotype such forms of care. In this way, the story demonstrates the value of a “cultural humility” rather than “cultural competence” model of health education. The language of “competence” has been criticized for implying mastery, and suggesting cultures are monolithic and knowable (Tervalon and Murray-García 1998). As Hooker and Noonan (2011) note, overemphasis on presumed cultural “differences” between patients and providers risks Othering patients. At its worst, this model can result in racist generalizations, like those in the since-retracted *Nursing: A Concept-Based Approach to Learning* (2014). In a textbox titled “Focus on Diversity and Culture: Cultural Differences in Response to Pain,” the authors make claims like “Hispanics may believe that pain is a form of punishment and that suffering must be endured if they are to enter heaven” and “[Native Americans] tend to be less expressive both verbally and nonverbally” (Jaschik 2017). Cultural humility, in contrast, demands awareness of healthcare’s power differentials and prioritizes communication styles that allow individual patients to guide encounters as “the physician relinquishes the role of *expert* to the patient” (Tervalon and Murray-García 1998, 121). With its generations of healers and depiction of an individual integrating biomedical and traditional epistemologies, “Remedies” illustrates that, although grounded in community, the cultural traditions that shape healing remain deeply individual.

Reading “Remedies” with health humanities students can facilitate such crucial conversations about the relationship between traditional and biomedical approaches to health. While “Remedies” is but one of many literary texts amplifying Latinx models of healing, there are practical and conceptual aspects of the story that make it particularly apt for classroom use. In addition to being freely available online, the text’s practical strengths include its short form, engaging narrative voice, and the numerous interviews Fajardo-Anstine has given about how her lived experience shapes her writing (Arreola 2024; Fajardo-Anstine and Kawakami 2020; Luna 2019). Its use of an adolescent narrator also makes it a relatable read for undergraduate students. When teaching “Remedies,” we began by asking students to share remedies they learned at home. After a period of reflective writing, students eagerly shared the healing practices they grew up with. As students compared remedies learned from parents and grandparents of Mexican, Filipino, Ghanaian, Slovakian, Indian, African-American, Jewish, Chinese, and other heritages, many were surprised by similarities across cultures as well as variations between families of shared backgrounds. Tervalon and Murray-García (1998) argue for the value of having future health professionals “think consciously about their own, often ill-defined and multidimensional cultural identities and backgrounds” (120). Reflecting on their home remedies underscores for students that we all have culturally-informed understandings of health that shape but don’t dictate our approach to healing. Furthermore, the act of sharing in class offered practice in approaching unfamiliar health practices with curiosity and humility.

“Remedies” also rejects a binary that pits the physical and emotional efficacy of traditional healing practices against biomedicine. Notably, though rooted in traditional knowledge, neem has also been the subject of scientific studies over the last few decades, as researchers seek to better understand its efficacy (Heukelbach et al. 2006; Mehlhorn et al. 2011; Thawornchaisit et al. 2012). The story itself reflects this porous boundary between traditional and biomedical approaches to health by offering a portrait of the ways people navigate multiple beliefs. Following this initial activity, we drew students into conversations about the relationship between such traditions and biomedicine by turning to a specific quote in which Clarisa reflects on her integration of multiple medical epistemologies: “For the most part, I stick to over-the-counter remedies. They are cleaner and work faster and come in packages with childproof lids. But every once in a while, when I get a real bad headache and the aspirin isn’t cutting it, I take slices of potatoes and hold them to my temples, hoping the bad will seep out of me” (85). In class discussions, students noted that this passage reflected the way they themselves navigated biomedicine, family remedies, and self-care practices. Thus, the story offers an important complement to frequently taught texts like *The Spirit Catches You and You Fall Down,* in which the seemingly intractable conflict between U.S. biomedicine and Hmong beliefs escalates to tragic heights. “Remedies” offers an antidote to such binary thinking and can get students talking about ways they might draw out and integrate their future patients’ beliefs–as well as their own.

Ultimately, “Remedies” encourages health humanities students invested in biomedical thinking to grapple with its limitations. As Ellis (2021) notes, interrogating the meanings traditional healers accrue through literary depictions “requires facing the terrifying reality that biomedicine fails us” (11). Attending to biomedicine’s shortcomings, including its dualistic approach to health, lack of emphasis on spiritual well-being, and individualist ethos, invites students to view it as just one of many models of healing that warrant respect–an especially important lesson for students planning to pursue healthcare careers. Reading “Remedies” affirms the value of students’ existing expertise and prepares them for clinical settings where providers and patients alike negotiate diverging models of health.

## Data Availability

N/A.
